# Unlocking the Potential of Collagenases: Structures, Functions, and Emerging Therapeutic Horizons

**DOI:** 10.34133/bdr.0050

**Published:** 2024-10-08

**Authors:** Zhen-Zhen Wang, Kang Wang, Ling-Feng Xu, Chang Su, Jin-Song Gong, Jin-Song Shi, Xu-Dong Ma, Nan Xie, Jian-Ying Qian

**Affiliations:** ^1^ School of Life Sciences and Health Engineering, Jiangnan University, Wuxi 214122, PR China.; ^2^ Cytori Therapeutics LLC., Shanghai 201802, PR China.

## Abstract

Collagenases, a class of enzymes that are specifically responsible for collagen degradation, have garnered substantial attention because of their pivotal roles in tissue repair, remodeling, and medical interventions. This comprehensive review investigates the diversity, structures, and mechanisms of collagenases and highlights their therapeutic potential. First, it provides an overview of the biochemical properties of collagen and highlights its importance in extracellular matrix function. Subsequently, it meticulously analyzes the sources of collagenases and their applications in tissue engineering and food processing. Notably, this review emphasizes the predominant role played by microbial collagenases in commercial settings while discussing their production and screening methods. Furthermore, this study elucidates the methodology employed for determining collagenase activity and underscores the importance of an accurate evaluation for both research purposes and clinical applications. Finally, this review highlights the future research prospects for collagenases, with a particular focus on promoting wound healing and treating scar tissue formation and fibrotic diseases.

## Introduction

Collagen, which is a diverse family of proteins, comprises approximately 30% of the total protein content and serves as the primary protein in the extracellular matrix (ECM) [1]. It is rich in Gly, Ala, Pro, and Hyp and low in Cys, Met, Tyr, and His, which together constitute its primary structure [2]. All collagen molecules share a common domain consisting of 3 polypeptide chains that fold into a unique triple-helical structure [3]. The collagen triple helix spontaneously aggregates and assembles into a network structure with a regularly staggered pattern, forming a collagen fiber bundle with a fairly crystalline structure (supramolecular structure) [4]. The van der Waals forces, hydrogen bonds, covalent cross-linking, spatial structure, and electronic effects among the 3 α-peptide chains give collagen high tensile strength [5]. Collagen can be hydrolyzed only under high-temperature, strongly acidic, and strongly basic conditions, which may destroy amino acids. However, collagenase can hydrolyze collagen under mild conditions with little destructive effect on amino acids and high specificity. Therefore, it is widely used in food processing, medical treatment, and tissue engineering applications [6].

Collagenase is an enzyme renowned for its ability to degrade collagen, a protein abundantly found in animals and microorganisms. It has the capacity to dismantle native fibrillar collagen under normal physiological conditions [6]. In an inaugural presentation at the first Collagenase Interdisciplinary Symposium, Ines Mandl of Columbia University emphasized the importance of collagen as a major component of the skin, tendons, cartilage, teeth, and bones. Collagenase promotes the degradation and resynthesis of collagen fibers, a process essential for normal development, wound healing, and tissue regeneration. In addition, collagenase is able to separate different types of cells from dense connective tissue, such as those found in skin, tendons, and bones. This enzymatic hydrolysis technique is gentler and more precise than mechanical methods, ensures the integrity of the cells and molecules, and is crucial for subsequent research. Clinically, collagenase is used to treat certain types of fibrosis; for example, collagenase injections have been used to treat Peyronie’s disease (PD) and to alleviate scarring in burn patients. In addition, collagenase promotes cell growth, migration, and differentiation by adjusting the composition and structure of the ECM, providing support for tissue repair and regeneration. Consequently, enzymes with the ability to degrade collagen play crucial roles in remodeling processes, as well as in laboratory studies of collagen and clinical applications involving collagen-rich structures [7]. Collagenase, an enzyme that digests peptide chains, specifically recognizes the Pro-X-Gly-Pro sequence and cleaves the peptide bond between an amino acid (X) and glycine (Gly). Commercial bacterial collagenases, which are predominantly obtained through the fermentation of *Clostridium histolyticum*, are complex mixtures comprising varying proportions of 2 main collagenase classes (class I and class II) along with other proteases. These enzymes are further classified into types I, II, III, IV, and V and hepatocellular-specific collagenases, each distinguished by their unique enzymatic activities and tailored for specific applications. The selection of the collagenase type should be tailored to the specific tissue type for separation and digestion (Table 1).

**Table 1.** Types and applications of commercial collagenases

Type	Component characteristics	Application	References
Type I	Relative average activity of multiple enzymes (including collagenase, *Clostridium* protease, trypsin activity)	Primarily employed for the cultivation of primary cells within epithelial tissue, liver, lung, adipose tissue, and adrenal tissue	[74,75]
Type II	Higher activity of *Clostridium* protease, compared with type I	Generally used to prepare primary cells from the heart, bone, liver, thymus, salivary gland, and cartilage	[76]
Type III	Low hydrolytic activity of secondary proteases	Preparation of primary cells from breast tissue	[77]
Type IV	Low pancreatic enzyme activity	Used for the preparation of islet cells	[78]
Type V	Partially purified type with higher collagenase and casein activities, but lower pancreatic enzyme activity	Suitable for islet preparation	[79]
NB	Relatively average amount of activity of multiple enzymes (including collagenase, *Clostridium* protease, neutral white enzyme activity)	Suitable for the separation of various cells in human and animal tissues, and has a wide range of effects	[80,81]

## Collagenase Sources

**Fig. 1.**
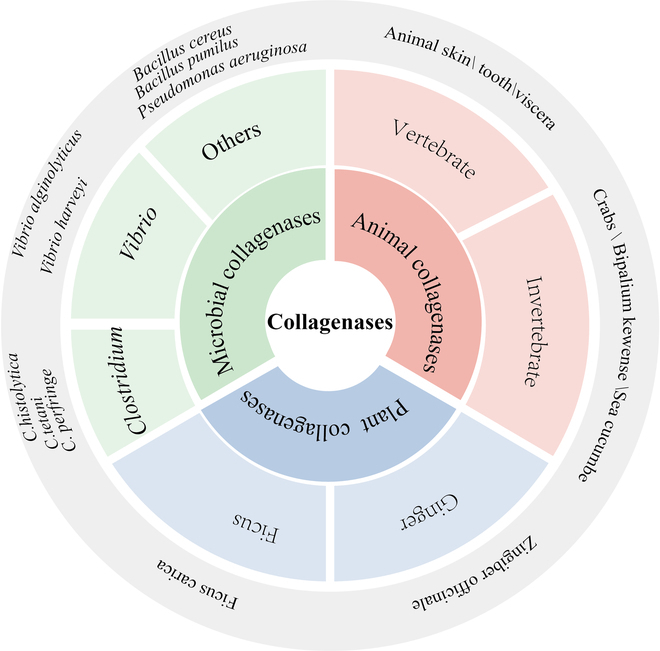
Classification of collagenases.

Collagenases have been identified in a wide range of organisms, including microorganisms, animals, and plants (Fig. 1). The primary focus of research is on matrix metalloproteinases (MMPs) and microbial collagenases.

### Plant collagenases

A single enzyme with protease and collagenase activities of 30 kDa was isolated from the crude extract of ginger root powder. In contrast to conventional collagenase, which targets a specific sequence in type I collagen, the ginger protease GP2 has the ability to hydrolyze natural type I collagen at identical sites within all 3 type I collagen chains [8]. Proteases derived from fig latex exhibit high specificity toward gelatin and collagen, as well as exceptional stability under varying temperature and pH conditions, thus having potential for diverse applications [9]. The utilization of plant-derived collagenases is currently limited compared with that of its microbial counterparts because of the intricate nature of plant systems.

### Animal collagenases

Animal collagenases, which are widely distributed among vertebrates, exhibit a distinct substrate preference by selectively cleaving the triple helix of collagen at approximately ^3^/_4_ of its molecular length from the X-Gly bond on the α chain. As members of the zinc metallopeptidase family, animal collagenases share collagenolytic activity with members such as MMP-1 (interstitial collagenase), MMP-8 (neutrophil collagenase), MMP-13 (collagenase-3), and MMP-18 (*Xenopus* collagenase-4). The presence of Zn^2+^ and Ca^2+^ ions contributes to stabilizing its tertiary structure [10].

Animal collagenases, which are part of the larger family of MMPs, are classified under the M10A subfamily in the peptidase database. These enzymes, often referred to as “matrixins”, are a group of zinc-dependent proteases that are crucial for the turnover and remodeling of the ECM. Collagenase is synthesized as an inactive precursor that undergoes activation through specific proteolytic cleavage events to become a fully functional enzyme. The inactivation of stromal proenzymes belonging to the M10A subfamily occurs through a mechanism known as the “cysteine switch” [11], wherein an interaction occurs between the conserved cysteine residue in the propeptide and the catalytic zinc ion, preventing key water molecules from binding. Due to their crucial role in maintaining proenzyme latency, cysteine residues can be activated by sulfhydryl blockers such as aminophenyluric acid acetate (APMA) by removing the N-terminal propeptide.

### Microbial collagenases

Currently, commercial collagenase is predominantly produced through the fermentation of *C. histolyticum*. Microbial collagenases belong to the peptidase family M9, which are metallopeptidases and can be further classified into the M9A and M9B subfamilies based on variations in amino acid sequences and catalytic functions (Table 2). Collagenases derived from *Clostridium* and *Vibrio* have been investigated more extensively than plant and animal collagenases.

**Table 2.** Collagenases from the peptidase family M9 in *Vibrio* and *Clostridium*. Data were obtained from the peptidase database (http://www.ebi.ac.uk/merops/).

Peptidase family	Bacterial genus	Strain	Molecular mass (kDa)	Amino acid number (aa)
M9A	*Vibrio* spp.	*Vibrio alginolyticus*	89.9	814
		*Vibrio harveyi*	89.7	814
		*Vibrio vulnificus*	80.9	715
		*Vibrio neptunius*	91.6	823
		*Vibrio owensii*	89.7	814
		*Vibrio parahaemolyticus*	89.8	814
		*Vibrio coralliilyticus*	91.4	823
		*Vibrio mimicus*	91.4	806
		*Vibrio cholera*	93.0	818
M9B	*Clostridium* spp.	*C. histolyticum*	126.2 (ColG) 116.4 (ColH)	1,118 (ColG) 1,021 (ColH)
		*Clostridium weizhouense*	141.4	1,244
		*Clostridium faecium*	128.3	1,135
		*Clostridium perfringens*	125.9	1,104
		*Clostridium sporogenes*	138.7	1,217
		*Clostridium haemolyticum*	111.8	966
		*Clostridium botulinum*	124.8	1,095

*Clostridium* utilizes collagenases, which represent one of the most efficient enzymes known to date for the degradation of native collagen, thereby facilitating nutrient uptake and host colonization. *Clostridium* collagenases are characterized as large multidomain proteins belonging to the metalloproteinase family [12]. According to the hydrolytic activity and stability of different substrates (collagen, gelatin, and synthetic peptides), as well as the amino acid sequences and protein structures of collagenases, *C. histolyticum* collagenases can be classified into 2 types: type I collagenase, which exhibits high activity toward natural collagen and gelatin but lower activity toward specific synthetic peptides, such as N-(3-[2-furyl]acryloyl)-Leu-Gly-Pro-Ala (FALGPA) and Pz-Pro-Leu-Gly-Pro-D-Arg (Pz peptides), and type II collagenase (displaying approximately one-third of the effectiveness against collagen and gelatin but higher efficacy against synthetic peptides) [13]. The *colH* gene encodes type I collagenase, whereas the *colG* gene encodes type II collagenase [14,15].

In addition to *Clostridium* and *Vibrio*, collagenases can also be produced by *Bacillus cereus* [16,17], *Bacillus dumilus* [18], *Clostridium perfringens* [19], and *Streptomyces* [20,21], among others. However, the crystal structures and mechanisms of action of most collagenases remain unresolved. Therefore, further investigations of collagenases and their coding genes are warranted to acquire sufficient data.

In discussing the different biological sources of collagenases, we recognize that each source has its own unique advantages and limitations. Plant collagenases, such as ginger protease, have attracted attention due to their unique substrate specificity and potential applications in food processing, but their commercial application may be limited by the complexity of plant systems and the cost of extraction and purification. Animal collagenases, especially those from mammals, are favored for their high substrate specificity and potential applications in tissue repair; however, their production costs are high, and the ethical issues involved cannot be ignored. Microbial collagenases, which are produced primarily through fermentation processes, have become commercially dominant due to their high yield and low-cost industrial production, but their application may be limited by endotoxin contamination and host immune responses. Future studies should focus on comparing the biochemical properties, substrate specificity, stability, and performance in various biomedical applications to gain a deeper understanding of these different sources of collagenases and provide guidance for further development and application of collagenases.

## Structures and Mechanisms of Collagenases

### Structures and mechanisms of MMPs

**Fig. 2.**
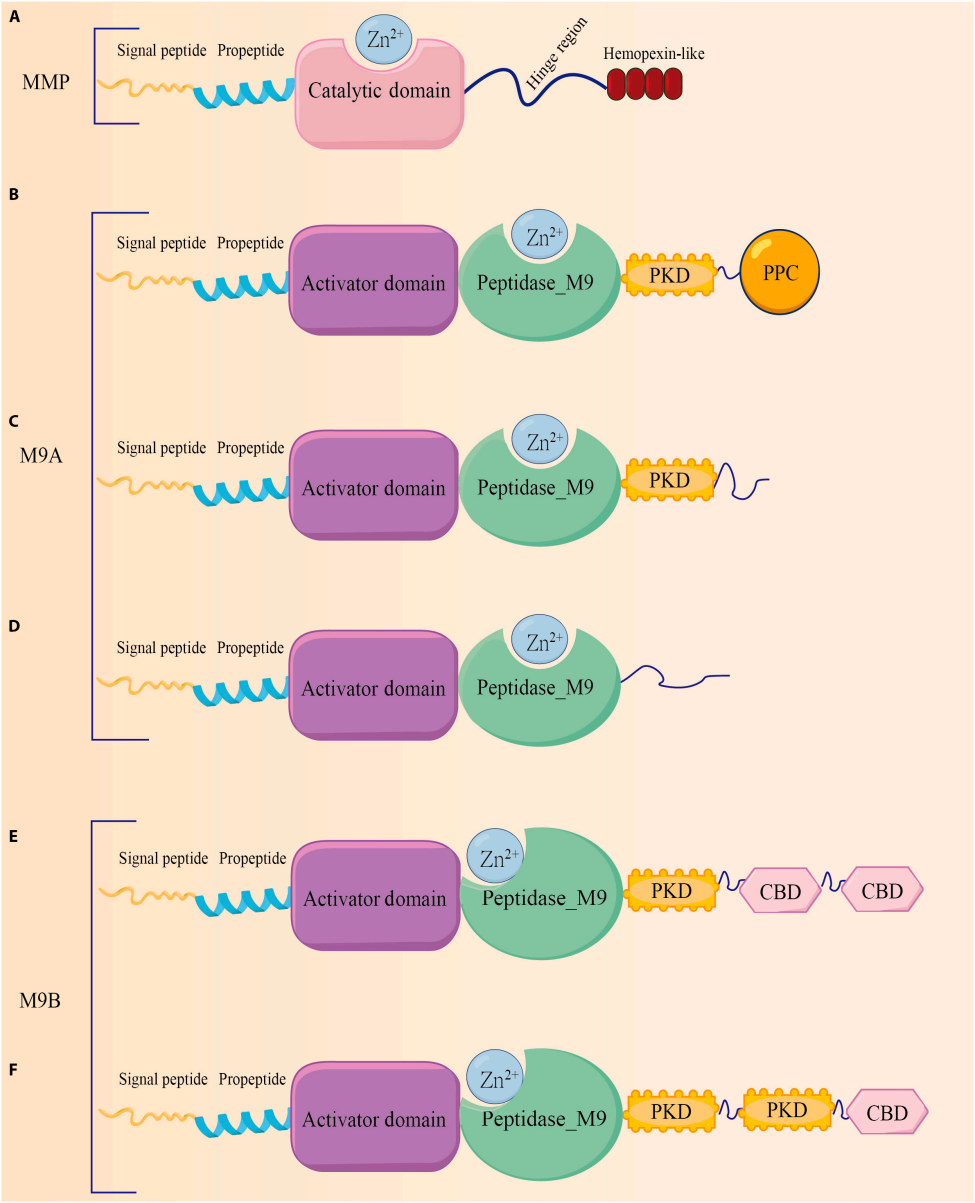
Structures of MMPs, M9A, and M9B. (A) Signal peptides direct the MMP to the secretory pathway or the plasma membrane insertion pathway. The propeptide maintains the stability of the proenzyme, and when this region is removed by exogenous enzymes, the MMP proenzyme is activated. The zinc-containing catalytic active region dominates enzyme-mediated catalysis, and the hinge region connects the catalytic and hemoglobin domains. (B) Domain structures of collagenase from *Vibrio parahaemolyticus*. (C) Domain structures of collagenase from *Vibrio alginolyticus*. (D) Domain structures of collagenase from *Vibrio mimicus*. (E and F) Domain structures of collagenases from *C. histolyticum* (ColG and ColH).

**Fig. 3.**
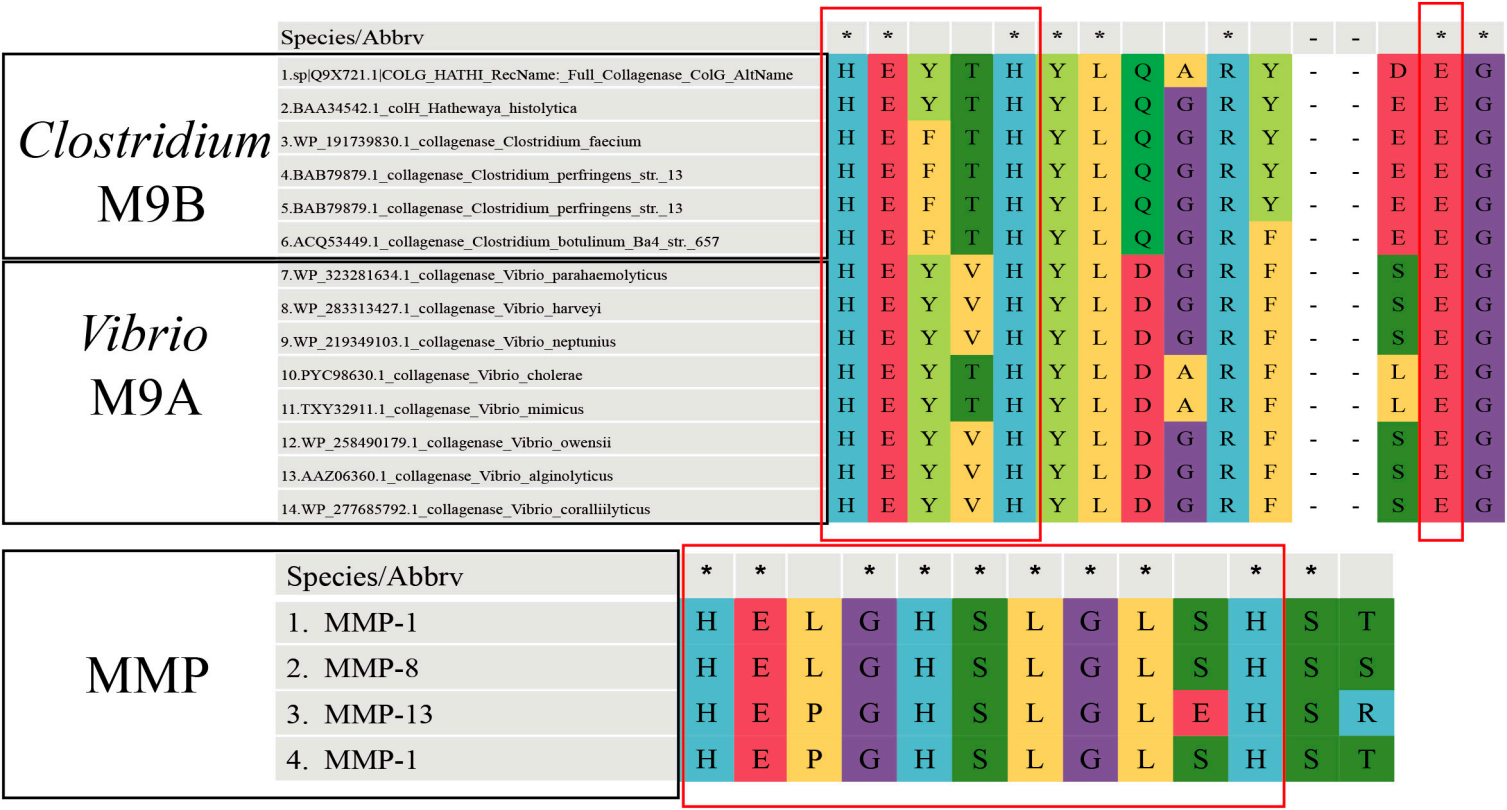
Multiple sequence alignment of the catalytic center of collagenases. The left box of M9 highlights the HEXXH motif (HEXXH+E), and the right box indicates the glutamate residue. The box of the MMPs shows the zinc-binding motif HEXXHXXGXXH in the catalytic domain.

Currently, mammalian MMPs, particularly MMP-1, play pivotal roles in the degradation of interstitial collagen and have been extensively investigated in terms of their structures and mechanisms. MMPs are characterized by a highly conserved motif comprising 3 histidines that coordinate with zinc at the catalytic site, as well as a conserved methionine residue positioned below the active site. Typically, an MMP consists of multiple domains, including a propeptide, a catalytic metalloproteinase domain, a linker peptide (hinge region), and a hemopexin (Hpx) domain (Fig. 2A) [22]. The catalytic domain of collagenases harbors the zinc-binding motif HEXXHXXGXXH (Fig. 3), which exhibits broad proteolytic activities; however, only full-length collagenases can cleave collagen with the assistance of the Hpx domain [23]. MMPs bind to and locally disrupt the triple-helical structure by hydrolyzing the C-telopeptide located at the outer periphery of the collagen fibril, thereby exposing the cleavage site and facilitating interactions between the enzyme and adjacent collagen triple helix. These enzymes subsequently hydrolyze peptide bonds and cleave fibrous collagen into characteristic ^3^/_4_ and ^1^/_4_ fragments [24–27]. A previous review provided a comprehensive analysis of the structural aspects of MMPs and elucidated the intricate molecular mechanisms underlying their physiological effects [28].

The synthesis of MMPs is directed by a signal peptide, which is a variable-length polypeptide sequence located at the N terminus of the protein. Its primary role is to facilitate the proper localization of newly synthesized MMPs within the secretory pathway. This peptide is subsequently removed by signal peptidase, resulting in the production of proMMPs or zymogens [29]. The primary role of the prodomain is to maintain MMP-1 in an inactive state following synthesis. It is characterized by a highly conserved sequence, known as the cysteine switch motif, which typically contains patterns such as PRCGXPD. This motif inhibits enzyme activity by forming coordination bonds with zinc ions at the active site within the catalytic domain, thereby preventing substrate binding [30]. The primary domain also contains 3 A-type helices, which form an elliptical ring structure in close proximity to the catalytic domain and a gap between its active sites. The first ring, positioned amidst helix 1 and helix 2, encompasses a protease-sensitive sequence referred to as the “decoy region”. This region undergoes specific cleavage under physiological conditions, such as pH variation, a unique ECM environment, or enzymatic action by other proteases. Consequently, this cleavage event destabilizes the prodomain and disrupts its interaction with the catalytic domain. Subsequently, disassembly occurs, allowing the exposure of the catalytic domain of MMPs to bind with substrates and thereby activate their proteolytic activity [31,32].

**Fig. 4.**
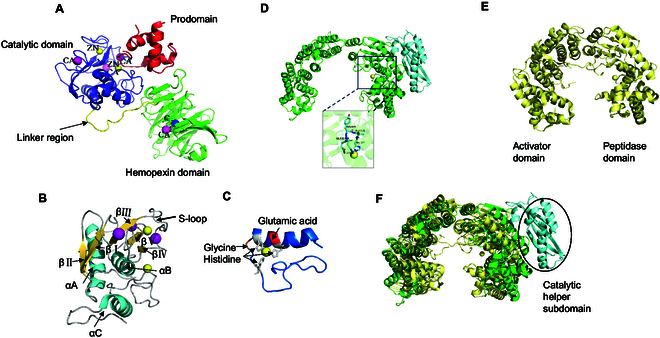
Collagenase crystal structures. (A) Ribbon representation of proMMP1. The MMP domains and inorganic ions are labeled and colored. (B) Ribbon representation of the catalytic site of proMMP1, with secondary structures colored and important segments labeled. (C) A ribbon representation of the active site of MMP1 is depicted, with the overall structure elegantly highlighted in a captivating teal hue. (D) Ribbon representation of ColG; the HEXXH zinc-binding motifs are labeled. (E) Ribbon representation of VhaC. (F) A comparison between the CM structures of VhaC (yellow) and ColG (green) is shown, and the catalytic helper subdomain of ColG is indicated by the circle.

After the removal of the prodomain, MMP-1 exhibits an exposed conformation and forms a collagen-binding site [33]. The catalytic domain, comprising approximately 170 amino acid residues, possesses a specific spatial arrangement necessary for enzymatic activity. Within the active site, 2 key features are present: a zinc ion essential for the catalytic function of the MMP and 3 histidine residues (typically located in the HEXXHXXGXXH motif, where H represents histidine, E denotes glutamate, G represents glycine, and X represents arbitrary amino acids) that coordinate with the zinc ion to constitute the core of the active site [34]. The shallow fissure for substrate binding is further divided into an upper N-terminal subdomain and a lower C-terminal subdomain, both of which are involved in the recognition and binding of substrates. In addition to the catalytic zinc ion at the active site, which is essential for catalysis, another zinc ion and 3 calcium ions contribute to the conformational stability of the protein structure. Multiple binding site pockets (S1, S2, S3, S1′, S2′, and S3′) within the active site play crucial roles in determining the substrate specificity and inhibitor binding of the MMP. Notably, among these pockets, the S1′ pocket holds particular significance in defining substrate specificity. A variable region known as the specific ring, which is composed of multiple amino acid residues that influence the ability of the MMP to bind and cleave specific substrates, is located in the upper region of the active site [29] (Fig. 4A and C).

The combination of these properties constitutes the structural foundation of the MMP catalytic binding site, facilitating its efficient recognition and cleavage of diverse substrates, as well as its involvement in ECM remodeling and various biological processes [26,35,36].

### Structures and mechanisms of microbial collagenases

In contrast to MMPs, which cleave the type I procollagen chain at a single site, microbial collagenases hydrolyze natural collagen at multiple sites and are predominantly classified into the M9, S1, S8, and unknown U32 peptidase families. Despite the identification of numerous microbial collagenases, their structures and mechanisms of action remain elusive.

#### *Clostridium* collagenases

*Clostridium* collagenases have a complex multidomain structure that is approximately 120 kDa in size and typically includes a prodomain, an N-terminal domain with catalytic zinc ions, multiple polycystic kidney disease-like domains, and one or more collagen-binding domains (CBDs) (Fig. 2E and F). The most representative *Clostridium* collagenases include ColG and ColH, which are derived from *C. histolyticum*.

The catalytic domain contains a thermolysin-like peptidase fold characterized by a mixed alpha and beta secondary structure that closely resembles leukotriene A4 hydrolase and F3 protease. This domain harbors a HEXXH Zn-binding motif, along with an additional glutamate residue positioned 28 to 30 amino acids downstream (Fig. 3D) [37]. In certain instances, the coordination of zinc ions may result in diminished affinity, thereby providing a potential mechanism for modulating enzyme activity [12]. The existing understanding of collagen identification and degradation by M9 collagenases mainly originates from the chew-and-digest concept introduced by Eckhard et al. [38], who elucidated the mechanism of *Clostridium* collagenase ColG. In this model, the process of collagen hydrolysis involves a transition between the open and closed states for the collagenase module (CM), which comprises an activator domain and peptidase domain. Initially, triple-helical collagen binds to the peptidase domain in its open state, followed by the closure of the CM to facilitate interactions with both the activator and peptidase domains. As a result, the peptide chain undergoes cleavage due to the unwinding of triple-helical collagen driven by entropy [39]. According to this model, the interaction between the activator domain and collagen in its triple-helical form, which is bound to the peptidase domain, exclusively occurs when the system is in a closed state, facilitating substrate unwinding by the peptidase domain.

#### *Vibrio* collagenases

A typical *Vibrio* collagenase comprises a signaling peptide, a CM encompassing an activation domain and a peptidase domain, a polycystic kidney disease-like domain (PKD-like domain), and a propeptidase C-terminal domain (PPC domain) [32] (Fig. 2B to D). Wang et al. [6] reported the identification of the *VhaC* gene, which encodes M9A collagenase from *Vibrio harveyi*. Researchers have employed cloning, expression, and purification techniques followed by x-ray diffraction analysis to determine its crystal structure. The analysis revealed that the CM forms a contracted saddle structure and lacks a catalytic helper subdomain. This structural characteristic distinguishes it from the CM structure of ColG, which contains an additional catalytic auxiliary subdomain essential for complete enzymatic activity (Fig. 3E and F). By generating mutants, the authors conducted further investigations into the functions of the PPC and PKD domains. The findings revealed a pronounced collagen-binding ability within the PPC domain, which plays a pivotal role in VhaC activity by facilitating enzyme localization to its substrate, collagen fibers, and this process promotes collagen degradation. Moreover, the PKD-like domain may act as an intermediary between the CM and the PPC domain in VhaC, thereby influencing the enzymatic activity of full-length enzymes on collagen fibers.

Unlike the chew-and-digest approach employed by ColG, in *Vibrio* collagenase VhaC, the activation domain initially recognizes triple-helical collagen, followed by subsequent cleavage of the peptidase domain during the closing movement of the CM. VhaC preferentially initiates proteolysis at the C-terminal nonhelical region of triple-helical collagen (C-telopeptide), resulting in the liberation of tropocollagen fragments that are further hydrolyzed into peptides and amino acids [6]. Nonetheless, a comprehensive understanding of the degradation mechanism and crystal structure of *Vibrio* collagenase requires further investigation.

## Acquisition of Collagenases

Collagenases are derived primarily from natural strains. Currently, numerous research groups have screened microorganisms that produce collagenases, including those sourced from the ocean [40,41], soil [42], tannery [43], and bone [44]. The majority of commercially available collagenases are obtained from *C. histolyticum*. Additionally, several heterologously expressed collagenases have been reported in the literature. For example, Zhu et al. [45] employed *Bacillus subtilis* to express genes encoding collagenases. In this section, we present the methods used for isolating and screening collagenase-producing microorganisms, as well as the expression of recombinant collagenases.

### Isolation and screening of collagenase-producing microorganisms

Although numerous microorganisms capable of producing collagenase have been documented, the methodology for screening collagenases remains a subject of controversy. Given that microorganisms can generate not only collagenase but also *Clostridium* protease, gelatinase, and other proteases, clear standards for both qualitative and quantitative assessments of microbial collagenase production must be established. The subsequent discussion will focus primarily on enumerating various collagenase screening methods employed by different research groups.

#### Qualitative methods for screening collagenases

Gelatin is derived from the denaturation of natural fibrin collagen and does not occur naturally. The hydrolysis of collagen into gelatin results in a mixture of amino acids connected by peptide bonds, with varying molecular weights [46]. Gelatin hydrolysis can be employed for the qualitative determination of collagenase activity. Numerous studies have documented the incorporation of gelatin as a supplementary component in various media formulations to screen for extracellular collagenase-producing microorganisms [42,47]. For example, solid media containing 0.5% glucose, 0.1% yeast extract, 0.2% KH_2_PO_4_, 0.7% K_2_HPO_4_, 0.02% MgSO_4_·7H_2_O, and 0.02% CaCl_2_·2H_2_O were supplemented with a concentration of 1.0% gelatin. Due to the gelatin hydrolysis activity of collagenases produced by microorganisms, a clear or transparent halo can be observed around microbial colonies. However, the visibility of the hydrolyzed zone may not be readily apparent on the medium in the plate. To increase visibility, the precipitation of proteins with trichloroacetic acid (TCA) can improve transparency in the hydrolyzed zone to increase visibility. Previous studies have shown that the addition of 35% TCA resulted in a distinct and well-defined gelatin hydrolysis zone [48] and precipitated proteins with an acidic mercury reagent (HgCl_2_) using the same principle. Researchers typically subject the obtained enzyme solution to digestion using goat/cow/fish skin, etc., followed by a secondary screen for bacteria exhibiting high collagenase activity to further confirm that the strains screened for the ability to degrade gelatin produce collagenase [49].

#### Quantitative methods for screening collagenases

Through qualitative screening in gelatin media, the enzymes are quantitatively assessed to identify the strain that presents the highest enzyme activity. Typically, collagen is employed as a substrate for reactions with fermentation-produced enzyme solutions to measure the quantity of free amino acids generated through substrate degradation. Although this method effectively quantifies collagenase activity, it is limited by lengthy reaction times and complex procedures. Alternatively, synthetic peptides such as FALGPA and Pz peptides are utilized as substrates. Compared with collagen, the degradation of these peptides occurs with shorter reaction times and markedly improved testing efficiency.

**Fig. 5.**
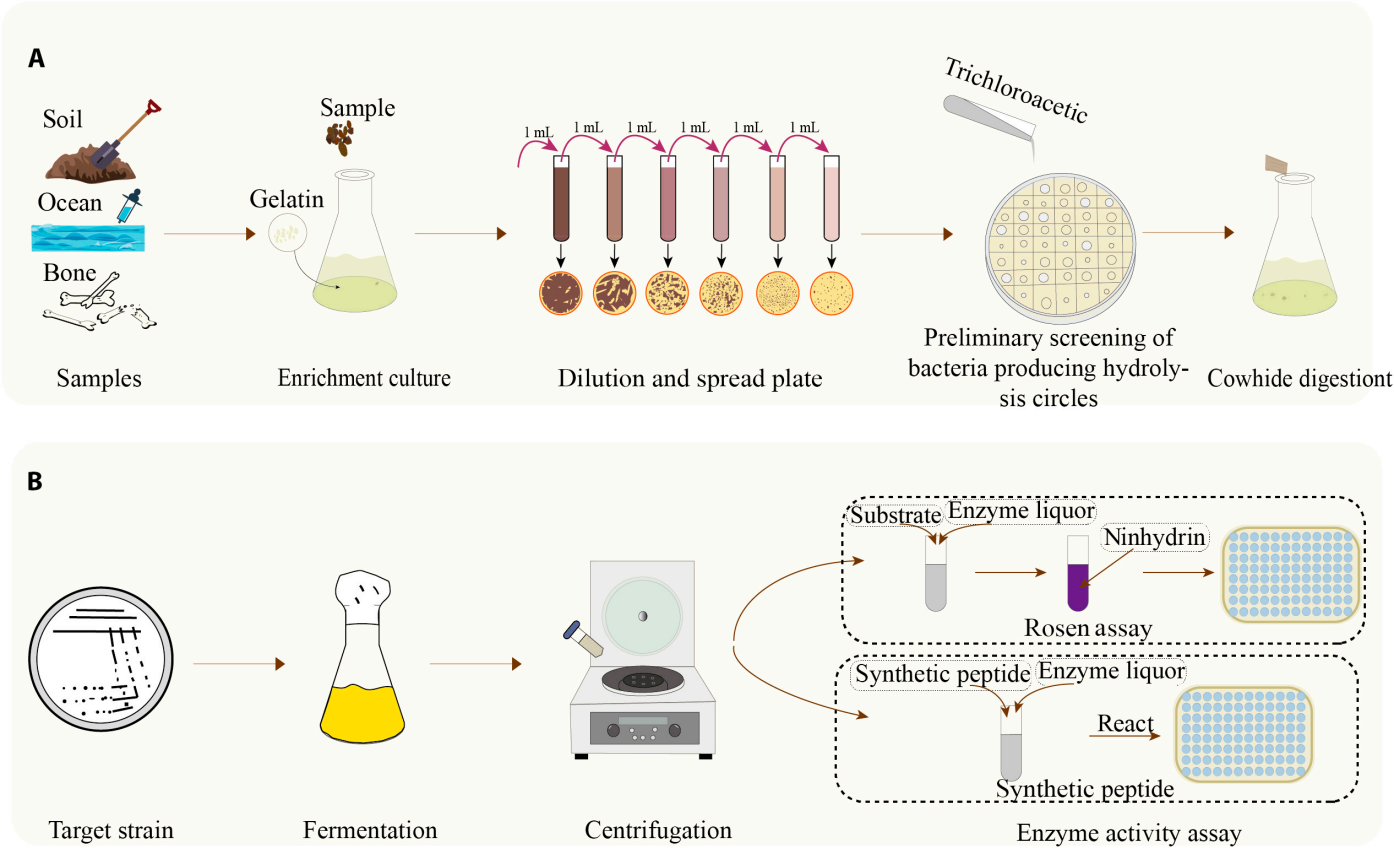
Schematic diagram of the methods used to screen wild-type strains for collagenase production. (A) Qualitative method for screening collagenase. (B) Quantitative methods for screening collagenase.

Occasionally, alternative methods for quantitatively detecting collagenase activity exist, such as the Azocoll assay, Hyp assay, electrophoresis assay, and viscosity assay. In summary, the qualitative screening method relies on observing the transparent halo generated by gelatin hydrolysis around a colony, which can be directly observed. Although gelatin is used as a substrate, it can reflect the activity of collagenase with strong specificity, but its sensitivity is weak because it relies on visual inspection, and it is not suitable for automated and large-scale screening. By measuring the amount of free amino acids produced by hydrolysis of specific substrates, quantitative screening methods provide greater sensitivity and accuracy. The specificity of natural collagen as a substrate is high, but the time needed to determine the enzyme activity is long and the operation is complicated, which limits the application of these methods in high-throughput screening. The reaction time for synthetic peptides as substrates is short and the methods are easy to operate, but they may not fully simulate the hydrolytic environment of natural collagen. Although isotope and fluorescence labeling methods have high sensitivity and are suitable for fine activity determination, their application in large-scale screening is limited by cost and technical requirements. Nevertheless, research into screening collagenases should focus on developing and optimizing high-throughput screening technologies that are fast, sensitive, accurate, and adaptable to automated operations. For example, screening platforms based on microfluidic technology that are capable of handling large numbers of samples while reducing the reaction volume and cost can be explored. In addition, combined with machine learning and artificial intelligence algorithms, rapid analysis and pattern recognition of screening data can be performed to further improve the accuracy and efficiency of screening and to more effectively identify and utilize collagenase-producing microorganisms (Fig. 5 and Table 3).

**Table 3.** Screening information for collagenase-producing microorganisms

Sample source	Strain	Molecular mass (kDa)	Applications	References
Deteriorated leather	*Penicillium* sp.	-	Used as a tool for treating leather industry waste	[82]
Hopped animal bone wastes	*Bacillus cereus*	38 kDa, metalloproteases	The hydrolysis of waste animal bones	[83]
Soil/sewage	*Pseudomonas* sp.	-	Broad substrate specificity and multiple commercially interesting applications	[49]
Soil and wastewater	*L. sphaericus*	110	Wound-healing effect of collagen membranes	[42]
*Melipona seminigra* bee	*Trichosporon* sp*.*	-	-	[84]
The green algal sample of *Codium fragile*	*Pseudoalteromonadaceae*	-	Collagen degradation and recycling in marine environments	[40]
Soils contaminated with fish wastes and traditional fermented fish products in Thailand	*Bacillus cereus* and *Klebsiella pneumoniae*	-	-	[48]

### Recombinant expression of collagenases

The application range of bacterial collagenase products is limited due to their complex compositions and low purity, and these products are derived primarily from freeze-dried cultures of *C. histolyticum*. Furthermore, the obvious variations in structure and function among bacterial collagenases necessitates a better understanding of their medical and biotechnological applications through heterologous expression of cloned genes. Heterologous protein expression is a straightforward and widely applicable experimental technique that enables the expression of target genes through the construction of recombinant expression vectors, transformation into host cells, and optimization of expression. This method is extensively employed for studying protein function without genetically manipulating the original host organism. Heterogeneous expression enables the production of large quantities of desired proteins, facilitating biochemical, structural, and functional analyses. Additionally, purification processes can be simplified by incorporating affinity tags such as His6 labels at either the N-terminal or C-terminal ends of recombinant proteins to facilitate the production of highly pure proteins [50].

#### Host selection

The most prevalent approach involves the utilization of prokaryotic heterologous expression systems, particularly *Escherichia coli*, for the production of proteins intended for commercial applications. *E. coli* possesses several advantages, such as rapid growth, a well-defined genetic background, ease of gene manipulation, efficient biomass accumulation, and straightforward scalability, making it an extensively investigated model organism. Nevertheless, codon bias may lead to reduced production of heterologous proteins within this organism. Regardless of the objective at hand, achieving biologically active proteins is a fundamental prerequisite for successful heterologous expression. In this context, careful selection of an appropriate host system tailored to specific protein requirements is highly important (Table 4).

**Table 4.** Summary of some heterologously expressed collagenases

Source	Host	Molecular mass (kDa)	Assay method	Activity	Purification method	Benefits	References
*C. histolyticum*	*Clostridium perfringens*	116	Pz peptide	1,100 ± 14 (1,070 ± 10) U/ml	Ammonium sulfate, ultrafiltration, and filtration through a nickel column	No endotoxin contamination. Greater stability of the plasmid and higher levels of protein production compared with *Bacillus subtilis.*	[50]
*Bacillus velezensis*	*Bacillus subtilis*	35.4	Rosen assay	1,145.16 U/ml	Ammonium sulfate, ultrafiltration, and filtration through a nickel column	The expression of collagenase in *B. subtilis* WB600 using a modified shuttle vector pP43NMK avoids the effects of harmful virulence factors.	[45]
*Bacillus cereus MBL13-U*	*E. coli BL21 (DE3)*	46	Rosen assay	64.99 U/ml	-	Efficient expression	[85]
*Grimontia hollisae*	*Brevibacillus pNY326 and HPD31-SP3*	62	FALGPA	9.39 U/mg	Size exclusion chromatography	-	[86]
*Grimontia (Vibrio) hollisae* 1706B	*Brevibacillus*	84	FALGPA	7.40 U/mg	Purified with a DEAE-Sepharose column with FPLC	As *Brevibacillus* is a bacterium that stains purple after Gram staining, this mechanism results in the production of modified proteins with minimal contamination from lipopolysaccharides. The recombinant enzyme derived from this system has the potential to facilitate the dispersion of human fibroblasts within a collagen gel, without any apparent harmful effects on cell viability (unpublished data).	[87]
*H. histolytica*	*S. cerevisiae*	116/126	FALGPA	0.015U/ml	-	The *yeast Saccharomyces* is widely utilized as a host for protein synthesis because it possesses a pathway for secreting proteins, is easily manipulated, and has the ability to withstand industrial conditions.	[88]

#### Optimization of collagenase expression

In general, heterologous expression often results in low protein expression levels in host cells, as well as potential errors during protein translational and folding processes. Various strategies can be employed to achieve optimal heterologous gene expression, including codon usage optimization for the recipient species, promoter modification optimization, utilization of Shine–Dalgarno sequences, increased messenger RNA (mRNA) stability, and a consideration of gene-splicing patterns. The degeneracy of codons allows each amino acid to be encoded by at least 1 codon and up to 6 different codons. Different species and organisms exhibit obvious variations in their preference for specific synonymous triplets. By replacing rare codons with optimal codons to increase codon usage bias, the expression of collagenase in *E. coli* is increased [51]. Currently, efforts toward efficient expression focus on investigating the functions and structures of collagenases due to their structural complexity and diversity.

## Determination of Collagenase Enzyme Activity

Enzyme activity is an essential parameter for the mining, creation, research, production, purification, and application of enzymes and enzyme preparations. Microbial collagenases use water-insoluble collagen or gelatin as substrates for hydrolysis, and their activity is determined by quantifying the amino acids released from the substrate. Collagenase activity can be affected by different factors, including the choice of substrate, its solubility, the amino acids produced by the substrate, and other conditions, such as pH, temperature, and the presence of metal ions (Table 5).

**Table 5.** Summary of collagenase activity assays

Method	Substrate	Enzyme activity	Benefit	Drawbacks	References
Rosen assay	Collagen	After a reaction at pH 7.4 and 37 °C (presence of calcium ions) for 5 h, the amount of peptides released from collagen was equal to the amount of color development of 1 μmol of leucine and ninhydrin, which was a CDU (one collagen digestion unit).	Qualitative and quantitative accuracy and reliability	The reaction time is long, and ninhydrin easily fades after color development	[40,89]
Synthetic peptide	FALGPA/Pz peptide	One FALGPA unit refers to the hydrolysis of 1 μmol of FALGPA per minute at 25 °C pH 7.5 (calcium ions present).	Rapid reaction, the substrate is stable and invariable, suitable for large-scale inspection	Does not directly reflect the hydrolytic activity of collagenase toward natural collagen	[86,90]
Electrophoresis assay	Gelatin	/	Rapid qualitative analysis	Unquantifiable	[86]
Azocoll assay	Azocoll	The light absorption value at A_520_ is increased by 1.0 per minute, which is defined as one unit of enzyme activity.	The operation is simple, the specificity is high, the colored hydrolysate is stable	Does not directly reflect the hydrolytic activity of collagenase toward natural collagen	[57]
Isotope labeling	^14^C-labeled collagen	Scintillation counting is performed to characterize enzyme activity, and collagenase activity is represented by the dpm value.	The sensitivity is high, suitable for in vivo testing	The equipment is expensive and produces radioactive contamination	[91]
Fluorescence labeling	FITC-collagen	/	Qualitative and quantitative accuracy and reliability	The substrate is expensive, the solvent affects the fluorescence intensity	[92,93]

### Rosen assay

Moore and Stein [52] initially proposed ninhydrin colorimetry for the quantitative analysis of the amino acid content in 1948. This method relies on the detection principle that amino acids react with ninhydrin under weakly acidic conditions to produce a blue–purple compound known as 1,2-indanedione (also referred to as Ruhemann’s purple). The absorption peak of this compound occurs at a wavelength of 570 nm, and within a specific range of color intensity (i.e., absorbance), it is directly proportional to the concentration of amino acids. Rosen [53] modified this technique by introducing a sodium cyanide–ninhydrin reagent, which was further adapted by researchers for measuring collagenase activity.

Collagen or gelatin, which serves as the substrate, is incubated with the enzyme for a specific duration, and the amino group liberated from collagen is subsequently quantified through ninhydrin color development. The traditional definition of enzyme activity is as follows: at 37 °C and under specific pH conditions, one unit of enzyme activity corresponds to the amount of enzyme required to produce 1 μmol of glycine or leucine per minute during collagen hydrolysis. For commercial enzymes, the Rosen assay is commonly employed, where enzyme activity is determined based on a reaction at pH 7.4 and 37 °C (in the presence of calcium ions) for 5 h. The quantity of peptides released from collagen should be equivalent to the color development resulting from 1 μmol of leucine and ninhydrin, which represents one unit [collagen digestion unit (CDU)] of product activity that must exceed 125 CDUs.

### Synthetic peptides

Synthetic peptides, such as FALGPA [54] and Pz peptide, contain Leu-Gly-Pro, which is the specific cleavage site of collagenase and has a higher reaction rate than other commonly utilized substrates. Additionally, the Rosen assay is time consuming (3 to 18 h), which may lead to collagen degeneration; however, this concern can be effectively mitigated by the use of synthetic substrates. The enzyme activity toward the synthetic peptide is denoted as 1 FALGPA unit, referring to the hydrolysis of 1 μmol of FALGPA per minute at pH 7.5 and 25 °C in the presence of calcium ions. Nevertheless, since collagenase specifically degrades the native fibril structure of natural collagen, measuring enzyme activity using synthetic short peptides as substrates does not directly reflect the hydrolytic activity of collagenase toward natural collagen. Therefore, a more accurate and reliable approach is to employ natural collagen as a substrate when screening the enzymatic activity of collagenases.

### Electrophoresis assay

MMPs bind and locally unwind the triple-helical structure of collagen, subsequently hydrolyzing the peptide bonds and cleaving fibrous collagen into characteristic ^3^/_4_ and ^1^/_4_ fragments, which can further be degraded by gelatinase and other nonspecific cathepsins; therefore, the enzymatic hydrolysis of collagen by MMPs can be qualitatively determined by electrophoresis. In nonreducing electrophoresis, a protease is reversibly bound by sodium dodecyl sulfate (SDS), temporarily loses its enzyme activity, and migrates on SDS–PAGE (polyacrylamide gel electrophoresis) gels containing collagen. After electrophoresis, the enzyme is eluted using buffer containing Triton X-100 to remove SDS, thereby restoring enzyme activity. Subsequent incubation under specific conditions allows for staining, where the digested region appears as a transparent band against a dark background [55,56]. This method enables a rapid qualitative analysis of collagenase activity.

### Other assays

Azocoll, an azo dye, can be specifically hydrolyzed by collagenase and a few other enzymes, enabling the characterization of collagenase activity through the measurement of the dye concentration in the reaction solution [57]. However, due to the availability of synthetic substrates suitable for measuring collagenase activity, this method is now less commonly employed. An alternative approach utilizing radiolabeled collagen substrates offers high sensitivity and broad applicability. Terato et al. [58] utilized ^14^C-labeled soluble collagen as a substrate to separate enzyme–substrate interactions and characterized enzyme activity through scintillation counting of the resulting supernatant. Nevertheless, this technique requires expensive equipment and is challenging to implement in standard laboratories while also generating radioactive waste that contributes to pollution.

When evaluating collagenase activity, selecting the appropriate assay method is crucial. Each approach has advantages and limitations and is suitable for different research and application needs. The Rosen assay is considered the gold standard for accurate qualitative and quantitative assessments but requires a longer reaction time, making it more suitable for detailed research rather than high-throughput screening. In contrast, synthetic peptides represent a rapid and stable alternative, and are ideal for large-scale applications where speed is crucial but may not fully represent natural collagen hydrolysis. The electrophoresis assay provides a quick qualitative assessment of enzyme activity, which is beneficial for preliminary screens. The Azocoll assay is highly suitable for educational and preliminary testing settings because of its simplicity and exceptional specificity. For studies prioritizing sensitivity, such as in vivo experiments, the isotope labeling method is preferred, despite the need for specialized equipment. Finally, fluorescent labeling methods, although qualitative, enable real-time monitoring of enzyme activity and prove invaluable in live-cell imaging and kinetic studies. Considering these factors when selecting an assay method ensures that meaningful results are applicable to the intended use case. Nonetheless, a rapid, precise, and cost-effective detection method holds immense significance in collagenase research.

## Applications of Collagenases

**Fig. 6.**
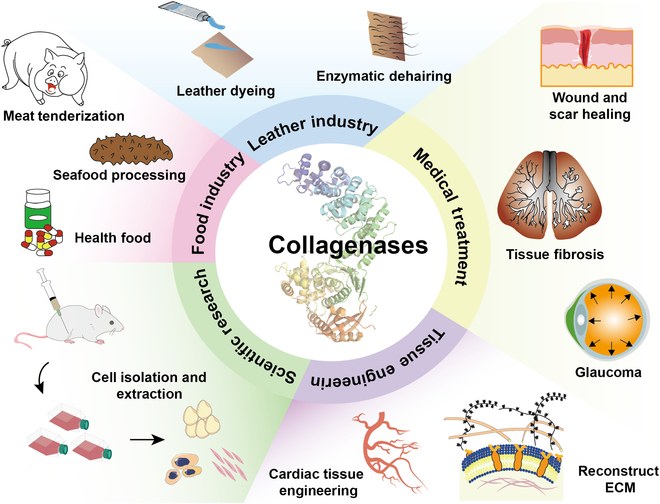
Applications of collagenases in medical sectors.

Collagenases have a broad range of applications, encompassing the fields of food processing, tannery operations, meat tenderization, and pharmaceutical compound synthesis. Most notably, they play a vital role in the medical sector by facilitating the treatment of burns, wounds, and scar tissue; organ transplantation procedures; PD management; and liver cirrhosis. Additionally, they can be utilized to improve blood purification methods, which can enhance diagnostic screening capabilities. Furthermore, these substances mitigate destructive fibrosis (Fig. 6).

### Scientific research

Tissues consist of diverse cells, and in the field of cellular and molecular biology research, the initial isolation of cells from specific tissue samples is imperative for understanding cell function or investigating the impacts of certain drugs. Collagenases have been extensively applied in the process of separating cells from mammalian fibrous tissues or delicate tissues, such as adipose stem cell and islet cell isolation and extraction [59,60]. Various cell culture techniques necessitate enzymatic digestion for cell separation, including the isolation of adipose tissue-derived stem cells (ADSCs), which are mesenchymal stem cells obtained from adipose tissue that are capable of inducing lipogenic and osteogenic differentiation. Type I collagen (Col1) serves as a major constituent of the ECM and is intricately intertwined with polysaccharide proteins to form a network structure that imparts mechanical strength. Adipose stem cells are typically isolated using enzymes, and different enzymes exhibit variations in their efficacy and differentiation potential; among these enzymes, collagenase is widely employed [61]. In clinical practice, the use of collagenase to separate islet cells for transplantation [62], the separation of adipose stem cells for treatment [63], and other methods have been reported (Table 6).

**Table 6.** Collagenases used for cell separation from related tissues

Separated tissue	Cell	Enzyme	Concentration	Result	References
Adipose tissue	Adipose-derived stem cells (ADSCs)	Collagenase I	0.1%–0.2%	The most commonly used enzyme for separating adipose-derived stem cells.	[59,94]
		MMP-12 (recombinant expression)	/	The ADSCs obtained exhibited remarkably similar morphological and phenotypical characteristics as cells isolated using collagenase I.	[95]
Pancreatic tissue	Islet cells	Collagenase V	0.5–2.5 g/ml	The islet cells were isolated effectively, but the use of collagenase alone resulted in low islet production.	[96]
		Liberase HI (collagenase I, II, and thermolysin enzyme)	According to the instructions	The most widely used commercially available enzyme for clinical islet isolation.	[60]
		Collagenase NB1/NP	According to the instructions	Compared to liberase HI, the volume of digestive tissue is reduced and the number of islets obtained is lower.	[97]
Liver	Hepatocytes	Collagenase VI	/	The protection of surface protein activity, especially mGluR5, was obviously enhanced in isolated rat hepatocytes.	[98]
		N-Acetylcysteine and liberase	According to the instructions	The efficiency of hepatocyte separation was obviously improved, and the production of living cells was increased.	[99,100]
		Collagenase V	0.5 mg/ml	It exhibits comparable efficacy to the liberase enzyme while providing superior cell viability.	[100]

### Tissue engineering

The application of collagenase in tissue engineering is focused primarily on the modeling and construction of the ECM environment, as well as the promotion of cell–ECM interactions [64]. Tissue engineering aims to develop biocompatible materials that can effectively replace or repair natural tissues by integrating principles from both biology and engineering. Collagenase plays a crucial role in degrading and reshaping natural ECMs to suit specific requirements for cell growth and tissue regeneration. For example, in cardiac tissue engineering, it is utilized to treat scar tissue formation following myocardial infarction, thereby facilitating neovascularization and cardiomyocyte implantation [65]. Moreover, collagenase can be employed to increase the biocompatibility and bioactivity of various natural biological materials, such as collagen and gelatin. These treated biomaterials can function as scaffolds for facilitating cell growth and enhancing cell attachment, proliferation, and differentiation. In investigations of cellular migration and invasion processes, such as cancer cell metastasis, collagenase can mimic the degradation behavior of cells during ECM traversal in vivo [66]. By regulating collagenase activity, examining the cellular response to ECM alterations becomes feasible. Collagenase enables the preparation of scaffolds with specific pore structures and mechanical properties. By adjusting the collagenase treatment conditions, the scaffold degradation rate can be controlled to match the tissue regeneration rates after implantation. For tissue damage or defect repair purposes, the localized application of collagenase promotes ECM degradation and regeneration while supporting damaged tissue repair and regeneration.

The application of collagenase in tissue engineering exemplifies its pivotal role in governing the composition and architecture of the ECM. By precisely modulating the activity and environmental conditions of collagenase, achieving the meticulous regulation of the cellular microenvironment becomes feasible, thereby providing a potent tool for advancing tissue engineering.

### Medical treatment

Collagenase selectively degrades cell membranes and the ECM and cleaves collagen. The purified form of collagenase has gained considerable recognition in the therapeutic management of tissue fibrosis, lumbar disc herniation, wound healing, and scar resolution [67,68]. Burns induce alterations in local cellular viability through chemical and mechanical mediators, leading to tissue necrosis. While burn-induced inflammation mitigates the infection risk, the persistence of debris, proinflammatory cytokines, and free radicals can exacerbate burn progression by promoting fibrotic changes and scarring [69,70]. Frederick et al. [71] assessed the efficacy of *Clostridium* collagenase in mitigating burn progression by employing a porcine model with brass comb burns. This study aimed to investigate the impact of *Clostridium* collagenase on the zone of stasis in burn wounds. The findings indicated that collagenase facilitates the early separation and loss of the epidermis, reduces necrosis, preserves and promotes blood vessel recovery and formation, limits apoptosis, and attenuates the infiltration of inflammatory cells. PD is a condition in which fibrous plaques form inside the penis, causing the penis to bend and potentially causing painful erections and sexual dysfunction. In 2013, the Food and Drug Administration (FDA) approved the use of *C. histolyticum* collagenase to treat PD. At present, clinical treatment with collagenase has long-term efficacy and relatively high safety and drug resistance. Based on the results of clinical studies, doctors and healthcare providers may consider it one of the recommended therapies for PD [72]. As a nonsurgical treatment option, *C. histolyticum* collagenase was approved by the FDA in 2010 for the clinical treatment of Dupuytren’s disease (DD), demonstrating the advantages of minimal invasiveness and rapid recovery [73].

## Outlook of Collagenases for Future Research

Currently, considerable debates surround the definition, classification, and identification of collagenase terms. Due to its protein nature, collagenase is highly influenced by factors such as temperature and pH during characterization experiments and in reactions. The process used to extract microbial collagenases often yields numerous side products, making industrialization challenging due to high costs. In the medical field, the application of microbial collagenases is crucial to ensure effective clinical monitoring for tracking patient recovery and recurrence rates. Most reported microbial collagenases lack structural or enzymatic characterization; hence, exploring the specificity, structures, and functions of these enzymes could improve our understanding of collagen degradation mechanisms while elucidating potential biotechnological applications.
